# Impact of loading dose β-lactam therapy on outcomes of KPC-producing *Klebsiella pneumoniae* bloodstream infections in non-ICU patients: a real world study

**DOI:** 10.1093/jac/dkag087

**Published:** 2026-03-05

**Authors:** Luisa Frallonardo, Giacomo Guido, Nicolò De Gennaro, Giuseppina De Iaco, Fabio Signorile, Marinella Cibelli, Antonio De Luca, Annunziata Ilenia Ritacco, Stefania Stolfa, Francesco Di Gennaro, Annalisa Saracino

**Affiliations:** Clinic of Infectious Diseases, Department of Precision and Regenerative Medicine and Ionian Area (DiMePRe-J), University of Bari Aldo Moro, Bari 70124, Italy; Clinic of Infectious Diseases, Department of Precision and Regenerative Medicine and Ionian Area (DiMePRe-J), University of Bari Aldo Moro, Bari 70124, Italy; Clinic of Infectious Diseases, Department of Precision and Regenerative Medicine and Ionian Area (DiMePRe-J), University of Bari Aldo Moro, Bari 70124, Italy; Clinic of Infectious Diseases, Department of Precision and Regenerative Medicine and Ionian Area (DiMePRe-J), University of Bari Aldo Moro, Bari 70124, Italy; Clinic of Infectious Diseases, Department of Precision and Regenerative Medicine and Ionian Area (DiMePRe-J), University of Bari Aldo Moro, Bari 70124, Italy; Clinic of Infectious Diseases, Department of Precision and Regenerative Medicine and Ionian Area (DiMePRe-J), University of Bari Aldo Moro, Bari 70124, Italy; Clinic of Infectious Diseases, Department of Precision and Regenerative Medicine and Ionian Area (DiMePRe-J), University of Bari Aldo Moro, Bari 70124, Italy; Clinic of Infectious Diseases, Department of Precision and Regenerative Medicine and Ionian Area (DiMePRe-J), University of Bari Aldo Moro, Bari 70124, Italy; Microbiology and Virology Unit, Department of Interdisciplinary Medicine, University of Bari ‘A. Moro’, Bari 70124, Italy; Clinic of Infectious Diseases, Department of Precision and Regenerative Medicine and Ionian Area (DiMePRe-J), University of Bari Aldo Moro, Bari 70124, Italy; Clinic of Infectious Diseases, Department of Precision and Regenerative Medicine and Ionian Area (DiMePRe-J), University of Bari Aldo Moro, Bari 70124, Italy

## Abstract

**Background:**

Bloodstream infections (BSIs) caused by KPC-producing *Klebsiella pneumoniae* (KPC-Kp) remain associated with high mortality, even outside intensive care units (ICUs). Optimizing early β-lactam exposure through a loading dose (LD) of ceftazidime–avibactam or meropenem–vaborbactam may improve outcomes, but evidence in non-ICU settings is limited.

**Methods:**

We conducted a retrospective single-centre study (January 2023–June 2025) including adult non-ICU inpatients with genotypically confirmed KPC-Kp BSI. The control group received standard dosing (ceftazidime–avibactam 2 g/0.5 g q8h or meropenem–vaborbactam 2 g/2 g q8h, both over 3 h). The LD group received either one standard dose infused over 30 min (Scheme A) or one standard dose + 50% infused over 2–3 h (Scheme B), followed by the standard regimen q8h. Primary outcomes were 7- and 30-day all-cause mortality; secondary outcomes included microbiological clearance ≤72 h, ICU transfer and/or vasopressor use, adverse events (AEs ≥ grade 2), hospital stay, and recurrence ≤30 days.

**Results:**

A total of 189 patients were included (140 controls, 49 LD). Groups were comparable in age (median 74 years), comorbidities (Charlson 5), and renal function (eGFR ≈45 mL/min/1.73 m²). LD was associated with faster blood-culture clearance (78% versus 55%; RR 1.26, 95% CI 1.03–1.61, *P* = 0.02) and lower ICU/vasopressor requirement (7.5% versus 23%; RR 0.60, 95% CI 0.29–0.93, *P* = 0.02). Median hospital stay was shorter (33 versus 37 days, *P* = 0.3). Thirty-day mortality was lower in LD (16.3% versus 21.0%; adjusted RR 0.62, 95% CI 0.39–1.29, *P* = 0.23), indicating a non-significant but clinically relevant trend. No increase in adverse events or nephrotoxicity was observed.

**Conclusions:**

In non-ICU KPC-Kp BSIs, a β-lactam loading dose regimen was associated with faster microbiological clearance, reduced ICU transfer and shorter hospital stay, without added toxicity. Mortality showed a non-significant trend towards improvement. Pragmatic LD strategies may enhance early β-lactam exposure where therapeutic drug monitoring (TDM) is unavailable. Prospective PK/PD-guided studies are warranted.

## Introduction

Carbapenemase-producing *Klebsiella pneumoniae* (Kp), particularly KPC-producing strains, represent one of the most challenging causes of healthcare-associated infections and sepsis.^1^ Despite the introduction of novel β-lactam/β-lactamase inhibitor combinations such as ceftazidime–avibactam and meropenem–vaborbactam, mortality from KPC-Kp bloodstream infections (BSIs) remains 20%–30% in non-ICU settings.^2,3^

The pharmacodynamic efficacy of β-lactams depends on the fraction of the dosing interval during which free drug concentrations exceed the pathogen’s minimum inhibitory concentration (fT > MIC). Achieving 100% fT > MIC during the first 24 h is essential to optimize bacterial killing and prevent early therapeutic failure. In clinical practice, standard intermittent dosing may lead to suboptimal early exposure, particularly in patients with augmented renal clearance, increased volume of distribution, or hypoalbuminemia—physiological alterations commonly observed among elderly, septic or comorbid individuals.^4^

A loading dose (LD) can mitigate early underexposure by achieving target β-lactam concentrations from the first administration.^5^ While PK/PD modelling strongly supports the use of LDs in critically ill patients, real-world evidence evaluating their clinical impact in non-ICU populations with KPC-Kp BSIs remains limited. Moreover, variability in patient characteristics, infection severity, and drug pharmacokinetics further complicates dose selection and may affect treatment response.^6^

Loading dose strategies represent a simple and readily implementable means to optimize early PK/PD target attainment for β-lactams when advanced monitoring tools are unavailable. Standardized loading dose regimens may therefore constitute a pragmatic and scalable approach to minimize the risk of early underexposure during the critical initial phase of KPC-Kp bloodstream infections. In clinical practice, there is no standardization regarding the applicability or dosing of the loading dose, particularly in patients with renal impairment. Currently, LDs are predominantly employed in critically ill ICU patients,^6,7^ whereas their role in sepsis that does not progress to shock is far less understood. In intensive care settings,^8^ loading doses are well established for β-lactams administered via extended or continuous infusion, yet their use with ceftazidime/avibactam or meropenem–vaborbactam in non-critically ill patients remains insufficiently studied, highlighting a significant evidence gap. Against this background, we aimed to assess whether an LD regimen of ceftazidime/avibactam or meropenem–vaborbactam could improve early clinical and microbiological outcomes, enhance pharmacodynamic target attainment during the critical first 24 h, and ultimately translate into better short-term survival among non-ICU adults with KPC-Kp BSI, addressing a key therapeutic gap in a population where early optimization of β-lactam exposure is seldom prioritized yet potentially decisive.

## Methods

### Study design and setting

A retrospective cohort study was conducted at a 1300-bed tertiary university hospital in Southern Italy between January 2023 and June 2025. The study protocol received approval from the Local Ethics Committee in October 2023 (protocol number 10/2023). Patient data were anonymized and managed in accordance with the Declaration of Helsinki and local data protection regulations.

### Eligibility criteria

Adult inpatients (≥18 years) with ≥1 positive blood culture for *K. pneumoniae* harbouring the KPC gene were included. Exclusion criteria: infective endocarditis; neutropenia <500/mm³; chronic renal insufficiency (eGFR <30 mL/min/1.73 m²); initiation of continuous renal replacement therapy (CRRT) ≤ 24 h after index culture; ICU transfer ≤24 h; or palliative care management only.

### Treatment exposure

Allocation to LD strategies was entirely based on treating physicians’ discretion, as no institutional protocol guided the use of loading doses during the study period. Two groups are acknowledged:

- Control group: ceftazidime/avibactam 2 g/0.5 g q8h (2–3 h infusion) or meropenem–vaborbactam 2 g/2 g q8h (3 h infusion).
- Two different loading dose (LD) strategies were used in our clinical practice:
- Scheme A consisted of one standard dose administered as a 30-minute infusion, followed by the standard maintenance regimen every 8 h.
- Scheme B consisted of one standard dose plus an additional 50% dose administered as a prolonged infusion over 2–3 h, followed by the standard maintenance regimen every 8 hours.

### Data collection

Demographics, infection source, Charlson Comorbidity Index (CCI), eGFR, inflammatory markers (CRP, PCT obtained prior to the initiation of antimicrobial therapy), polymicrobial infection, and time-to-active therapy (≤12 h versus >12 h) were recorded.

### Microbiology workflow

Blood cultures were processed using the BACT/ALERT VIRTUO system (bioMérieux, Marcy-l’Étoile, France). Positive bottles were subcultured on standard media, and species identification was performed by matrix-assisted laser desorption/ionization time-of-flight mass spectrometry (MALDI-TOF MS; Bruker Daltonics, Bremen, Germany). Antimicrobial susceptibility testing was carried out by broth microdilution and interpreted according to EUCAST clinical breakpoints (version 2023).

Carbapenemase production was screened using a lateral-flow immunochromatographic assay and confirmed by polymerase chain reaction detecting *bla*KPC. MIC values for ceftazidime–avibactam and meropenem–vaborbactam were determined using gradient diffusion strips (Liofilchem, Italy), with discordant or high-end values validated by broth microdilution. For each episode, only the index bloodstream isolate was considered for MIC analysis.

## Outcomes

Primary outcomes were 7-day and 30-day all-cause mortality. Secondary outcomes included microbiological clearance within 72 h, ICU transfer and/or vasopressor use, hospital length of stay, recurrent infection ≤30 days, and adverse events ≥ grade 2 (including KDIGO ≥1 nephrotoxicity and *C. difficile infection*).

Nephrotoxicity was graded according to the Kidney Disease: Improving Global Outcomes (KDIGO) criteria, with ≥ stage 1 considered significant (serum creatinine increase ≥0.3 mg/dL within 48 h or ≥1.5× baseline within 7 days). *Clostridioides difficile* infection was defined by compatible clinical findings plus a positive stool toxin assay or PCR.

## Statistical analysis

Continuous variables were expressed as medians [IQR] and compared using the Mann–Whitney U test. Categorical variables were compared by χ² or Fisher’s exact test.

Adjusted analyses were performed using inverse probability of treatment weighting (IPTW) derived from a logistic regression model including age, sex, Charlson Index, infection source, eGFR, MIC, time-to-active therapy (≤12 h, defined as the administration of an antimicrobial regimen with confirmed *in vitro* activity against the isolated KPC-Kp strain based on subsequent susceptibility testing), SOFA score, and Pitt bacteraemia score.

Risk ratios (RRs) were estimated using Poisson regression with robust standard errors, and weighted Cox proportional hazards models were applied for survival analysis. Balance between groups after weighting was verified using standardized mean differences (SMD <0.1).

A two-tailed *P* < 0.05 was considered statistically significant. Analyses were performed using Stata 17.0 (StataCorp, College Station, TX). Missing data were minimal and handled by complete-case analysis.

## Results

Among 189 included patients, 49 (26%) received a LD regimen (Scheme A *n* = 28, Scheme B *n* = 21). Median age was 74 years [IQR 67–81]; 57% were male. Both groups had high comorbidity (Charlson Index 5 [4–7]) and moderate renal impairment (median eGFR 45 mL/min/1.73 m²). Infection sources were predominantly urinary (38%) and abdominal (25%). The median SOFA score was 5 [3–9] in the control group versus 5 [3–7] in the loading-dose group (*P* = 0.47). Similarly, the Pitt bacteraemia score showed median 2 [1–5] versus 2 [1–4], respectively (*P* = 0.88), indicating no differences in initial clinical severity.

Baseline characteristics are shown in Table 1.

**Table 1.** Baseline characteristics of the study population

	Control (*n* = 140)	Loading dose (*n* = 49)	*P* value
Age, years [median (IQR)]	74 (67–81)	73 (65–80)	0.62
Male sex	81 (58%)	28 (57%)	0.88
Charlson Index	5 (4–7)	5 (4–7)	0.91
eGFR (mL/min/1.73 m²)	45 (32–68)	48 (35–71)	0.49
Peak CRP (mg/L)	199 (150–305)	205 (142–295)	0.33
Peak PCT (ng/mL)	11.1 (2.9–30.3)	9.7 (2.3–29.4)	0.42
SOFA score	5 (3–9)	5 (3–7)	0.47
Pitt score	2 (1–5)	2 (1–4)	0.88
Source: urinary	53 (38%)	18 (37%)	
Abdominal	34 (24%)	13 (27%)	
Catheter	31 (22%)	10 (21%)	
Indeterminate	22 (16%)	7 (15%)	0.85
Polymicrobial infection	27 (19%)	8 (16%)	0.68
Time-to-active therapy ≤12 h	127 (91%)	46 (94%)	0.59
Antibiotic: Ceftazime/avibactam	88 (63%)	30 (61%)	
Meropenem/vaborbactam	52 (37%)	19 (39%)	0.77

## Clinical outcomes and survival

At 7 days, mortality was 15.0% (21/140) in the control group versus 8.2% (4/49) in the LD group (adjusted RR 0.64, 95% CI 0.33–1.12, *P* = 0.09).

At 30 days, mortality was 21.0% (29/140) versus 16.3% (8/49) (adjusted RR 0.62, 95% CI 0.39–1.29, *P* = 0.23). Blood-culture clearance within 72 h was significantly higher in LD (78.0% versus 55.0%; RR 1.26, 95% CI 1.03–1.61, *P* = 0.02), and ICU transfer and/or vasopressor use was lower (7.5% versus 23.0%; RR 0.60, 95% CI 0.29–0.93, *P* = 0.02). Hospital stay was shorter (median 33 [21–43] versus 37 [24–49] days, *P* = 0.3). Rates of recurrent infection (12.2% versus 13.0%), adverse events (14.3% 12.1%), nephrotoxicity, and C. difficile infection were comparable (Table 2). No discontinuations occurred due to drug-related toxicity.

**Table 2.** Clinical outcomes and safety

	Control (*n* = 140)	LD (*n* = 49)	Adjusted Effect (95% CI)	*P* value
7-day mortality	21 (15.0%)	4 (8.2%)	0.64 (0.33–1.12)	0.09
30-day mortality	29 (21.0%)	8 (16.3%)	0.62 (0.39–1.29)	0.23
Blood-culture clearance ≤72 h	77 (55%)	38 (78.0%)	1.26 (1.03–1.61)	0.02
ICU transfer and/or vasopressors	32 (23%)	4 (7.5%)	0.60 (0.29–0.93)	0.02
Hospital stay, days [median (IQR)]	37 (24–49)	33 (21–43)	—	0.3
Recurrent infection ≤30 day	18 (13.0%)	6 (12.2%)	0.95 (0.40–2.26)	0.90
AEs ≥ grade 2	17 (12.1%)	7 (14.3%)	1.18 (0.52–2.67)	0.69
KDIGO ≥1 nephrotoxicity	9 (6.4%)	4 (7.3%)	1.27 (0.41–3.94)	0.68
C. difficile infection	3 (2.1%)	1 (2.0%)	—	0.96

**Figure 1.**
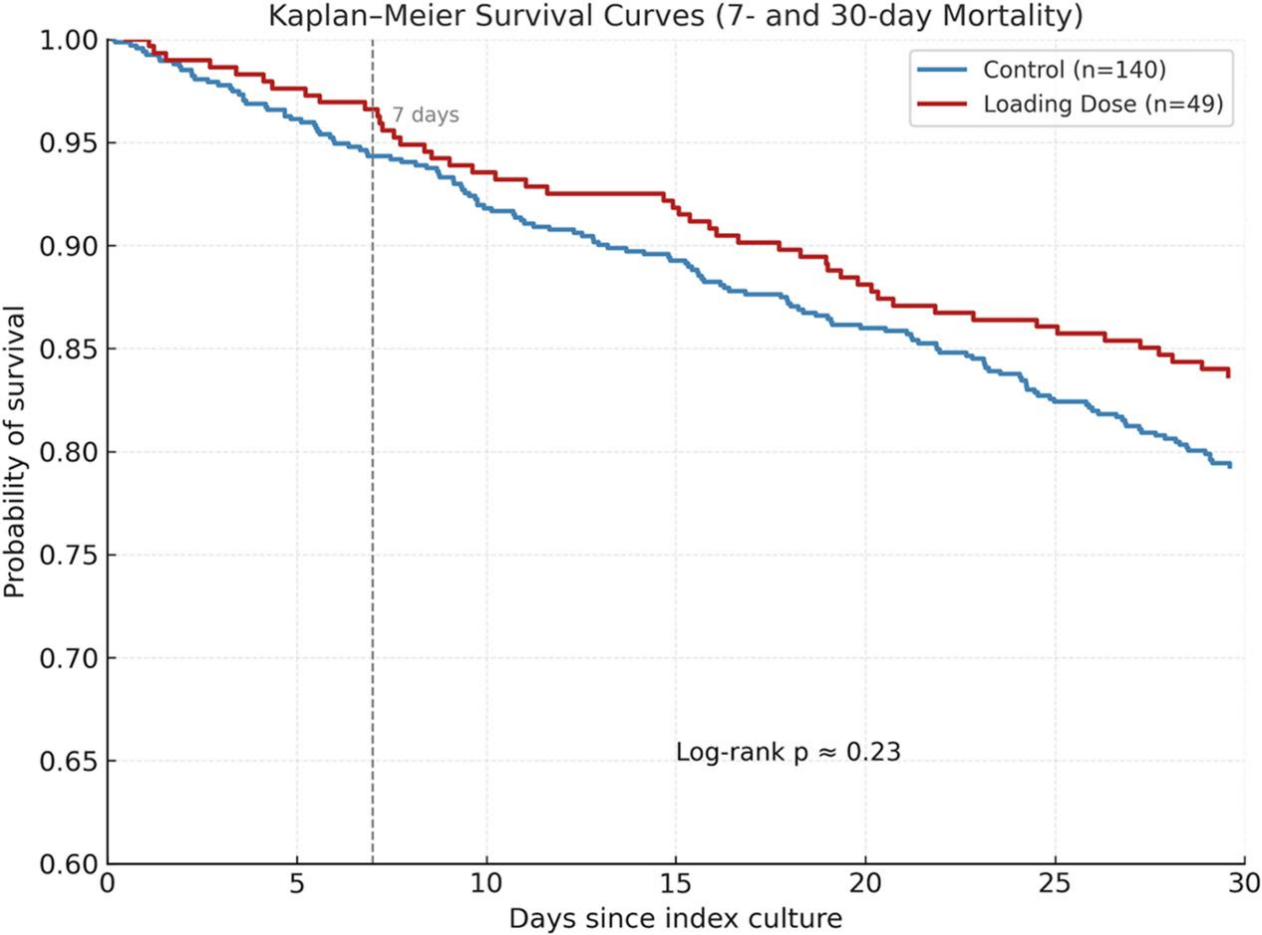
Kaplan–Meier survival curves at 7 and 30 days in patients with KPC-producing *Klebsiella pneumoniae* bloodstream infection treated with standard versus loading-dose β-lactam regimens.

In Figure 1, Kaplan–Meier analysis showed an early divergence of survival curves between the loading-dose and control groups, with lower cumulative mortality in the LD arm at both 7 and 30 days.

## Discussion

This real-world study suggests that implementing a β-lactam loading-dose strategy may enhance both early microbiological clearance and clinical stability in non-ICU patients with KPC-Kp bloodstream infections. To the best of our knowledge, no previous clinical study has specifically evaluated loading-dose strategies of ceftazidime–avibactam or meropenem–vaborbactam in non-ICU KPC-Kp BSIs.

Prior analyses in ICU cohorts^8^ focused mainly on prolonged infusion or renal-adjusted dosing, leaving the question of early exposure optimization unresolved. Our data therefore address a critical gap by exploring LD effects in a clinically distinct, non-critical population.

Although loading-dose strategies are traditionally emphasized in critically ill patients,^4^ several characteristics of non-ICU populations may likewise predispose to early β-lactam underexposure. Many patients with KPC-Kp bloodstream infections are elderly, multimorbid, or exhibit subclinical organ dysfunction that alters drug distribution and clearance, while others may experience transient episodes of augmented renal clearance during the early inflammatory phase.^7,8^

These factors, although less pronounced than in ICU settings, can still compromise initial pharmacokinetic target attainment. Notably, current guidelines^9,10^ provide no specific recommendations regarding the use of loading doses for ceftazidime–avibactam or meropenem–vaborbactam in non-critically ill patients, leaving a therapeutic gap in a population where early antimicrobial optimization may nevertheless be clinically relevant. Our findings therefore support the rationale for considering a loading dose even outside the ICU, particularly when rapid stabilization and early microbiological control are desirable.

For β-lactams, which exert time-dependent antibacterial activity, optimal PK/PD targets are achieved by maintaining plasma concentrations within a defined therapeutic range with minimal fluctuations.^11^ This concept is particularly evident in critically ill patients, in whom deep pathophysiological alterations frequently compromise adequate drug exposure.^12,13^

Population pharmacokinetic studies consistently show that extended (typically ≥3 h) or continuous infusions, administered after an appropriate loading dose, enhance the probability of achieving PK targets compared with standard intermittent infusions.^14^

Although PK/PD modelling consistently supports early target attainment with loading doses, clinical evidence outside intensive care settings remains limited^15,16^

To date, no international guidelines provide specific recommendations on loading dose strategies for ceftazidime–avibactam or meropenem–vaborbactam in non-ICU settings,^17,18^ further underscoring the originality and clinical relevance of our pragmatic approach, particularly in settings where therapeutic drug monitoring is not available.^19,20^

In this framework, our findings contribute novel data indicating that LD regimens may confer meaningful benefits even in patients who are not critically ill.

The LD approach was associated with a significantly higher likelihood of 72-h culture clearance, a reduced need for ICU escalation, and a modestly shorter hospital stay, without compromising safety or increasing adverse events. Although mortality at 7 and 30 days was lower in the LD group, differences did not reach statistical significance, likely reflecting residual clinical frailty, comorbidities, or other unmodifiable prognostic factors.

Although the differences in mortality did not reach statistical significance, the magnitude of the effect and the early separation of survival curves suggest a potentially clinically relevant signal. The lack of statistical significance is likely attributable to the limited sample size and the high burden of comorbidities and frailty in the study population, which may have reduced the statistical power to detect differences in mortality outcomes despite an apparent early benefit. Furthermore, mortality at 7 and 30 days was evaluated as all-cause mortality. In a population characterized by advanced age and a high burden of comorbidities, mortality may have been influenced by factors unrelated to the index infection or microbiological failure. This may explain why the observed microbiological advantage did not fully translate into a survival gain. The biological rationale is clear: achieving therapeutic β-lactam concentrations from the first administration mitigates the early underexposure caused by expanded distribution volume, altered renal function, or systemic inflammation.^16^

Early attainment of 100% fT > MIC is critical for bacterial killing and stabilization. The improvement in 72-h clearance strongly supports this pharmacodynamic hypothesis.

Our findings are consistent with prior PK/PD modelling studies^21^ and real-world analyses suggesting that optimized β-lactam dosing, including prolonged or loading infusions, reduces clinical failure in carbapenem-resistant Enterobacterales infections.

No increase in nephrotoxicity, *C. difficile* infection, or adverse events was observed with LD regimens, supporting the tolerability of higher initial β-lactam exposure even in elderly and in patients with moderate renal impairment. Importantly, no patients discontinued therapy because of drug-related toxicity, further supporting the safety of loading-dose β-lactam regimens in this population. From a clinical perspective, a pragmatic approach—such as administering one standard dose plus 50% over 2–3 h or a rapid first dose over 30 min—may be particularly useful in settings without therapeutic drug monitoring or during empirical therapy for suspected KPC-Kp BSI. Although the log-rank test did not demonstrate a statistically significant difference (*P* = 0.23), Kaplan–Meier analysis revealed the widest separation of survival curves within the first week, temporally aligned with faster microbiological clearance, underscoring the potential early clinical advantage of LD therapy. Groups were well balanced with respect to comorbidities, renal function, infection sources, inflammatory markers, and time to active therapy, thereby reducing potential confounding and enhancing the interpretability of outcome differences. The analysis was restricted to patients treated with strict monotherapy using ceftazidime–avibactam or meropenem–vaborbactam. Patients who received concomitant adjunctive antimicrobial agents (including fosfomycin, tigecycline, or colistin) were excluded in order to minimize confounding effects and mitigate bias related to combination therapy, thereby allowing a more accurate assessment of the independent impact of each β-lactam regimen on clinical outcomes.

However, we acknowledge some limitations that should be considered when interpreting these findings: this is a retrospective single-centre design, possible residual confounding despite IPTW adjustment, variability in MIC testing, and limited sample size for subgroup analysis (Scheme A versus B, ceftazidime–avibactam or meropenem–vaborbactam). In the absence of TDM, it was not possible to ascertain the adequacy of drug exposure in relation to the intended PK/PD targets.

Patients with chronic renal failure were excluded from the study, a methodological choice that may restrict the generalizability of the findings to broader clinical populations. Nevertheless, accumulating evidence indicates that dose reduction of ceftazidime/avibactam according to renal function in patients with KPC-Kp BSI is independently associated with increased mortality.^7^

The results may not be extrapolatable to institutions with differing local epidemiology, particularly those with a higher prevalence of VIM or NDM producers or documented ceftazidime–avibactam resistance.^13^ Given the limited sample size and the study’s focus on early PK/PD optimization rather than head-to-head comparison of LD strategies, no formal outcome comparison between the two LD schemes or across different MDROs was performed. No formal power analysis or sample size calculation was performed, given the retrospective and exploratory design of the study

In conclusion, in this real-life non-ICU cohort with KPC-producing *Klebsiella pneumoniae* bloodstream infections, the use of a loading-dose regimen of ceftazidime–avibactam or meropenem–vaborbactam was associated with faster microbiological clearance, reduced ICU transfer, and shorter hospitalization, without increased toxicity. Mortality improvement remained a non-significant trend. Pending prospective PK/PD-guided confirmation, a LD regimen followed by maintenance q8h may represent a rational, safe, and readily implementable in antimicrobial strategies to optimize early β-lactam exposure outside the ICU.

While results are encouraging, the observational design and limited sample size for Scheme A/B subgroups necessitate confirmation in multicenter or prospective PK-guided trials.
